# Supplementary material for: Genomic surveillance of multidrug-resistant Klebsiella in Wales reveals persistent spread of Klebsiella pneumoniae ST307 and adaptive evolution of pOXA-48-like plasmids
Source: Microb Genom. 2023 May 25;9(5):mgen001016. doi: 10.1099/mgen.0.001016 (PMC10272877; doi:10.1099/mgen.0.001016)
Supplement: Supplementary material 3 [file mgen-9-1016-s003.pdf]

Supplementary Information

Genomic surveillance of multidrug-resistant *Klebsiella* in Wales reveals persistent spread of *K. pneumoniae* ST307 and adaptive evolution of pOXA-48-like plasmids

Sophia David[1], Massimo Mentasti[2], Kirsty Sands[3,4], Edward Portal[4], Lee Graham[5], Joanne Watkins[5], Catie Williams[5], Brendan Healy[6], Owen B. Spiller[4], David M. Aanensen[1], Mandy Wootton[2] & Lim Jones[2]

Supplementary Table 1. Metadata, assembly statistics and genotyping results for 540 short-read sequenced *Klebsiella* isolates included in this study.
[Provided in Excel spreadsheet]

Supplementary Table 2. Metadata and hybrid assembly statistics for 39 long-read sequenced *Klebsiella* isolates included in this study.
[Provided in Excel spreadsheet]

Supplementary Table 3. URLs for interactive Microreact projects containing phylogenetic analyses for each *Klebsiella* species (those with ≥5 isolates only) together with all metadata and genotypic data.

Species	Microreact URL
K. pneumoniae	https://microreact.org/project/aSJqpc9MZVZcurTWtoadW8-k-pneumoniae-phw-n421
K. aerogenes	https://microreact.org/project/dsACmepLnijN2GKy9dr29s-k-aerogenes-phw-n33
K. michiganensis	https://microreact.org/project/8UDw4RarAEnVkMu4jDzCe5-k-michiganensis-phw-n27
K. variicola	https://microreact.org/project/4Ctdt1YhyrPcei6cswD4iw-k-variicola-phw-n23
K. oxytoca	https://microreact.org/project/f5w1sF46NFkExbHTLgDiNm-k-oxytoca-phw-n17
K. quasipneumoniae	https://microreact.org/project/aER2kfzsHKbin1ZWBvwU3y-k-quasipneumoniae-phw-n11
K. grimontii	https://microreact.org/project/jfHhJfsHs244sxb6ZqNziA-k-grimontii-phw-n5

Supplementary Table 4. Conjugation frequencies determined from mating cultures comprising ST307 donor isolates harbouring a $bla_{OXA\text{-}48\text{-}like}$-encoding pOXA-48-like plasmid and a recipient *K. pneumoniae* or *E. coli* strain.
[Provided in Excel spreadsheet]
